# Weather and electrical demand and consumption data of a small Mexican community

**DOI:** 10.1016/j.dib.2023.109977

**Published:** 2023-12-16

**Authors:** Milagros Santos-Moreno, Jorge Ángel González-Ordiano, J. Emilio Quiroz-Ibarra, D.A. Perez-DeLaMora, Jaime Mizrahi-Cojab, Emilio Román-Sánchez, José Pablo Montero-Cantú, Lazaro Bustio-Martinez

**Affiliations:** a Instituto de Investigación Aplicada y Tecnología, Universidad Iberoamericana, Ciudad de México, México; b Departamento de Estudios en Ingeniería para la Innovación, Universidad Iberoamericana, Ciudad de México, México

**Keywords:** Energy data, Weather data, Smart grid

## Abstract

The development of novel technologies to mitigate the effects of climate change through Smart Grids requires energy related data. Unfortunately, this type of data is not always available in Mexico, especially from non-large urban areas and at the household level. Therefore, we present a dataset that contains electrical demand and consumption time series of 5 households within a small community in Mexico, at various resolutions, as well as weather data. The electrical demand is given in 15 min resolution, while the electrical consumption is presented in both hourly and daily resolutions. The data is contained within 15 separate .csv files; one for each household's resolution. In turn, the weather data is given in two .csv files (for outdoor and indoor variables, respectively) that together contain 24 meteorological variables measured in a 5 min resolution that is not always consistent. The dataset comprises of two separate folders that contain either the electrical demand and consumption files or the weather files. This dataset could aid in the development of novel smart grid methods and algorithms that might be able to push the energy transition in Mexico and other developing countries forward.

Specifications Table

**Table:** 

Subject	Energy Engineering and Power Technology
Specific subject area	Energy informatics
Data format	Raw
Type of data	Time series (Table)
Data collection	The electrical demand and consumption data were measured using the *Emporia Vue Smart Energy Monitor, with 50A circuit sensors*. The sensors were installed in the electrical panels of each of the studied households and connected to their WiFi network. The sensors upload the information of the households’ power demand and consumption to an online platform from which we can download the data. Furthermore, meteorological conditions were measured using the *Ambient Weather WS-2000 WiFi OSPREY Solar Powered Wireless Weather Station*; both its outdoor and indoor measuring units were installed in one of the monitored households. The weather station system measured 24 different variables and uploaded them to an online platform that allowed us to easily download them.
Data source location	Town: Tepanco de López (18.5538, -97.5605) Region: Puebla State (19.03793, -98.20346) Country: Mexico.
Data accessibility	Repository name: Electrical demand/consumption and climatological data of a small Mexican community. Data identification number: 10.17632/vsjtbzjttb.4 Direct URL to data: doi.org/10.17632/vsjtbzjttb.4

## 1 Value of the Data

• The presented dataset consists of the electrical demand and consumption time series of 5 households within a small community in the Mexican State of Puebla called Tepanco de López. This type of information is valuable, since time series of household power demand and consumption in resolutions as the ones presented are not easily accessible in Mexico.
• The time series data can be used to develop, for instance, energy forecasting methods and grid control algorithms fine-tuned to the realities of a small community.
• This information is also of value, as it may give researchers a better understanding of the time series data stemming from households within a small Mexican community.
• The dataset also contains weather information measured directly in the community. Having both power and weather data can aid researchers simulate more realistic scenarios of the renewable energy integration in such a community.
• The presented weather and electricity demand and consumption data can also be used to simulate the power consumption of buildings, to study how to reduce energy consumption, and to better understand the energy dynamics in small Mexican communities.

## 2 Data Description

The dataset includes information about electrical demand/consumption and meteorological data within a small community in the State of Puebla, presented in two different folders named *electrical_demand_consumption*, and *weather.* As their names suggest, the first folder contains the electrical demand and consumption information, while the other contains the corresponding climatological variables. In the following paragraphs, a geographical and climatological description is presented, as well as a more thorough description of the contents of each data folder.

### 2.1 Geographical and Climatological Overview

**Fig. 1:**
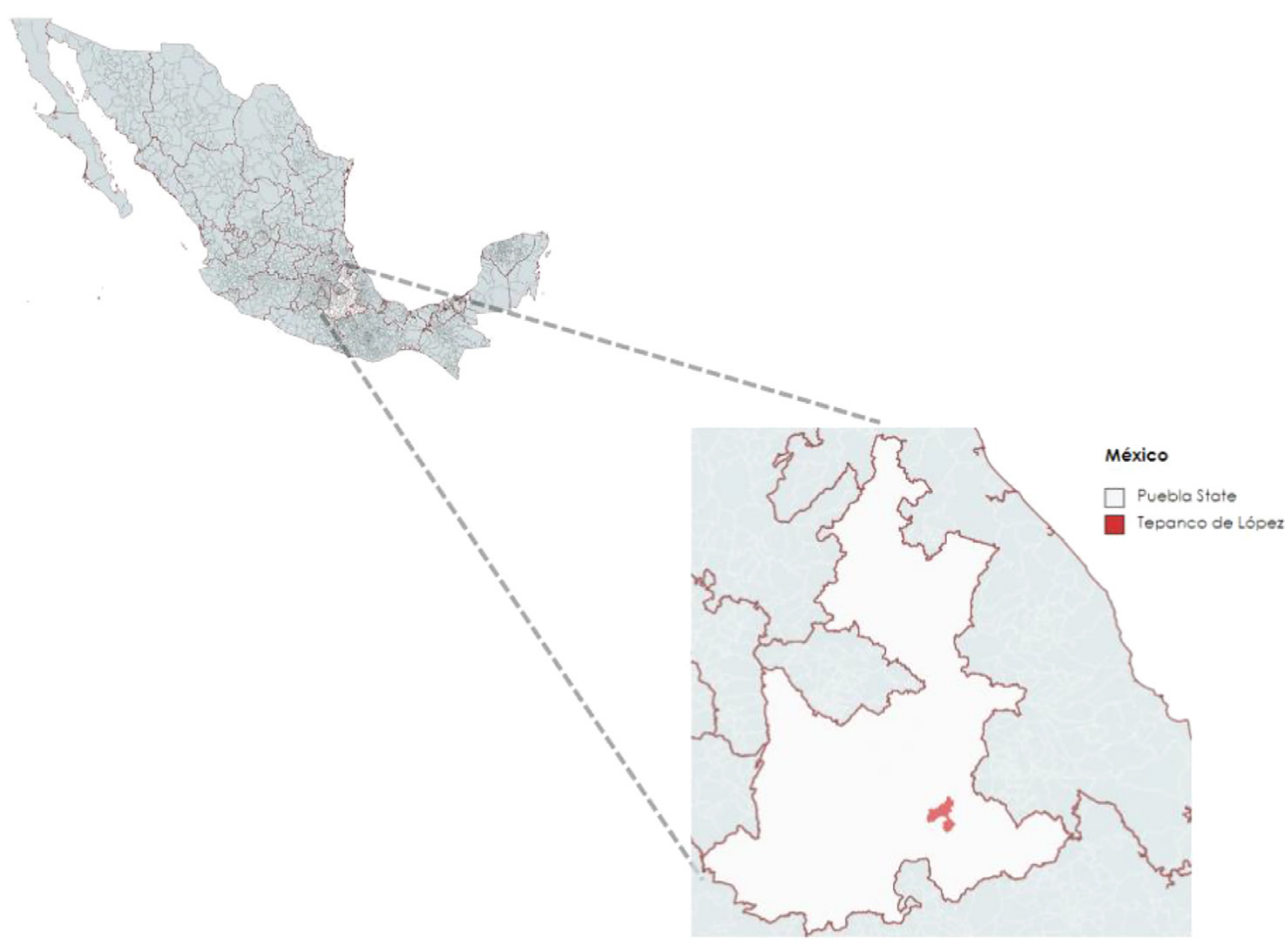
Municipality's location in the state of Puebla, Mexico^1^.

First, a general description of the municipality of Tepanco de López and its climatological information is presented. Tepanco de López is a municipality with an extension of 207.95 km^2^ located in the southern part of the state of Puebla, as shown in Fig. 1 [1]. The region exhibits three types of climates: sub-humid with summer rainfalls, tempered semiarid and warm semiarid. In terms of precipitation, the municipality is classified as Region III, with an annual average of 747.98 mm [2]. Finally, the annual average temperature ranges from 14 °C to 20 °C [3].

### 2.2 Electrical Demand and Consumption Time Series

Within the *electrical_demand_consumption* folder there are fifteen .csv files containing time series of the electrical demand and consumption of five different households. Note that they are labeled as houseteh1, houseteh6, houseteh7, houseteh8, and houseteh10^2^. Within the folder there are three .csv files for each household; one containing the 15 min electrical demand measurements (in kW), another containing the 1 h electrical consumption values (in kWh), and a final one containing the daily electrical consumption measurements (in kWh). For the sake of clarity, these files have their resolution included in their names, for instance, the files containing information of the *i*th household would be named as *housthei_15min.csv, housthei_1h.csv*, and *houstehi_1day.csv*. Table 1 contains the label of each file and the description of its contents.

**Table 1:** File names and content description of the files contained within the *electrical_demand_consumption* folder.

File name	Description
houseteh1_15min.csv	15 min demand measurement for House 1
houseteh1_1h.csv	Hourly consumption measurement for House 1
houseteh1_1day.csv	Daily consumption measurement for House 1
houseteh6_15min.csv	15 min demand measurement for House 6
houseteh6_1h.csv	Hourly consumption measurement for House 6
houseteh6_1day.csv	Daily consumption measurement for House 6
houseteh7_15min.csv	15 min demand measurement for House 7
houseteh7_1h.csv	Hourly consumption measurement for House 7
houseteh7_1day.csv	Daily consumption measurement for House 7
houseteh8_15min.csv	15 min demand measurement for House 8
houseteh8_1h.csv	Hourly consumption measurement for House 8
houseteh8_1day.csv	Daily consumption measurement for House 8
houseteh10_15min.csv	15 min demand measurement for House 10
houseteh10_1h.csv	Hourly consumption measurement for House 10
houseteh10_1day.csv	Daily consumption measurement for House 10

Every file contains only two columns, the first one is named Time, in which the date and time are given using the dd/mm/yyyy HH:MM format, and a second one named after the unit of the values contained within the file (i.e., kW or kWh). Therefore, we can argue that data is given in the form of a time series. In principle, every file contains measurements from May 2022 to May 2023, nevertheless, since the different sensors were installed at different moments, their number of measurements might be different. Note that the household 7 files contain large amounts of missing values, due to an unexpected event that complicated the internet connection of that specific sensor. For all other households, except household 10, the only missing values are present on March 12th, 2023, due to the start of the daylight-saving time. Bear in mind that most of Mexico—including the state of Puebla—no longer observes the daylight-saving time (abolished in October 2022). It is important to mention that all missing values are labeled with a -1; these values do not stem from the sensors, but rather were added by us to make the identification of the missing values easier. For the sake of understanding, Table 2 contains the start and the end time of for every file measurement, as well as the number of data and missing values within it.

**Table 2:** Start time, end time, number of measurements and number of missing values in every file within the *electrical_demand_consumption* folder.

File name	Start time	End time	Measurements	Missing values
houseteh1_15min.csv	21/05/2022 14:15	01/06/2023 00:00	36,040	4
houseteh1_1h.csv	21/05/2022 15:00	01/06/2023 00:00	9010	1
houseteh1_1day.csv	21/05/2022 00:00	01/06/2023 00:00	377	0
houseteh6_15min.csv	21/05/2022 13:30	01/06/2023 00:00	36,043	4
houseteh6_1h.csv	21/05/2022 14:00	01/06/2023 00:00	9011	1
houseteh6_1day.csv	21/05/2022 00:00	01/06/2023 00:00	377	0
houseteh7_15min.csv	19/05/2022 20:00	01/06/2023 00:00	36,209	16,882
houseteh7_1h.csv	19/05/2022 20:00	01/06/2023 00:00	9053	3476
houseteh7_1day.csv	19/05/2022 00:00	01/06/2023 00:00	379	190
houseteh8_15min.csv	21/05/2022 14:45	01/06/2023 00:00	36,038	4
houseteh8_1h.csv	21/05/2022 15:00	01/06/2023 00:00	9010	1
houseteh8_1day.csv	21/05/2022 00:00	01/06/2023 00:00	377	0
houseteh10_15min.csv	21/05/2022 16:00	01/06/2023 00:00	36,033	449
houseteh10_1h.csv	21/05/2022 16:00	01/06/2023 00:00	9009	45
houseteh10_1day.csv	21/05/2022 00:00	01/06/2023 00:00	377	2

### 2.3 Time Series Representation

**Fig. 2:**
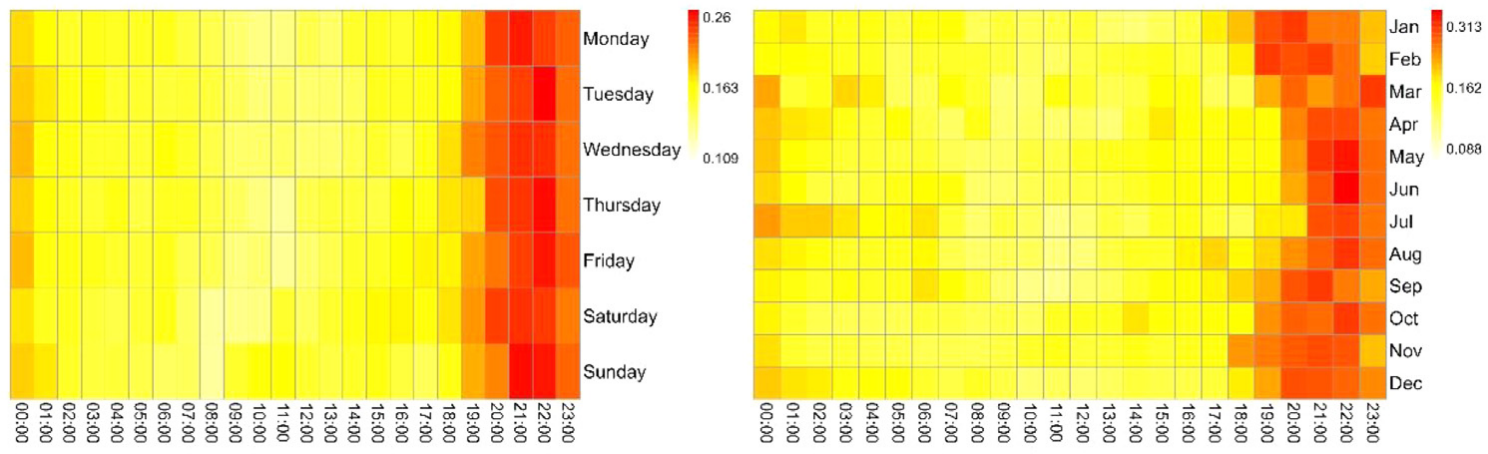
Heat Maps for electrical consumption (KWh) in household 1.

**Fig. 3:**
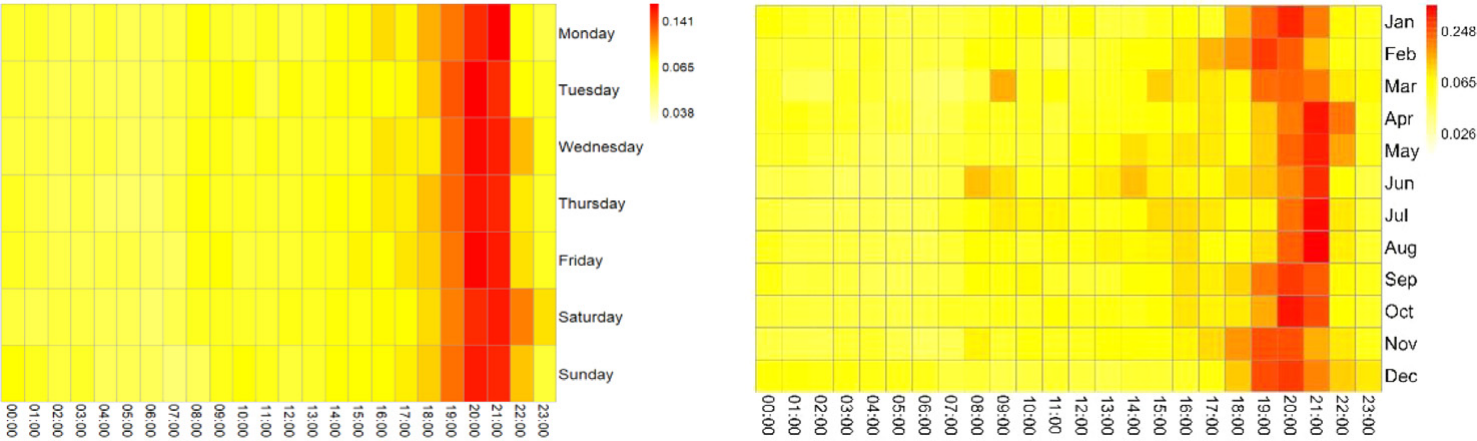
Heat Maps for electrical consumption (KWh) in household 6.

**Fig. 4:**
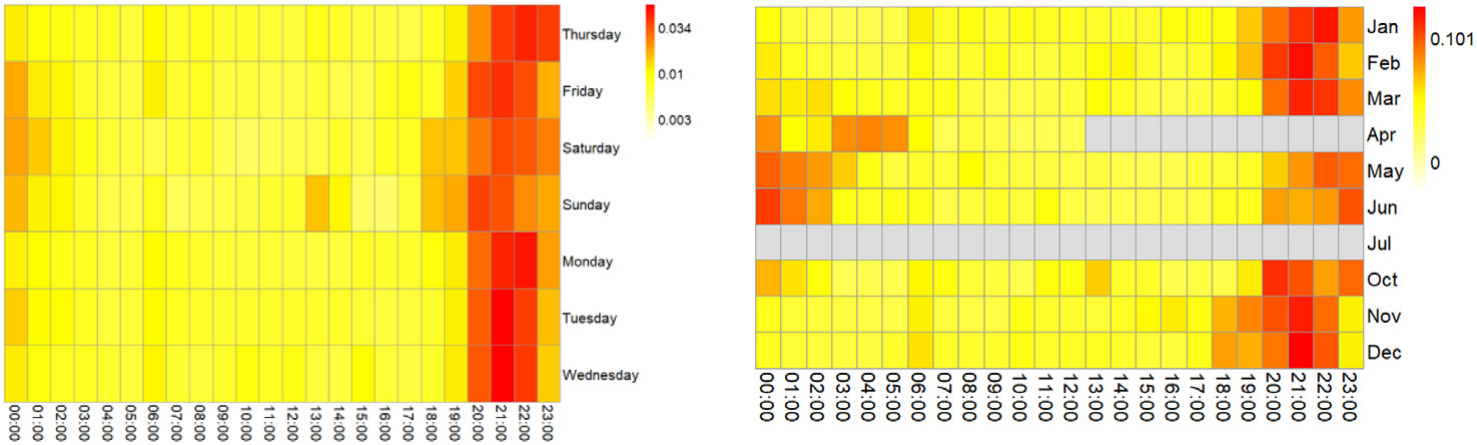
Heat Maps for electrical consumption (KWh) in household 7.

**Fig. 5:**
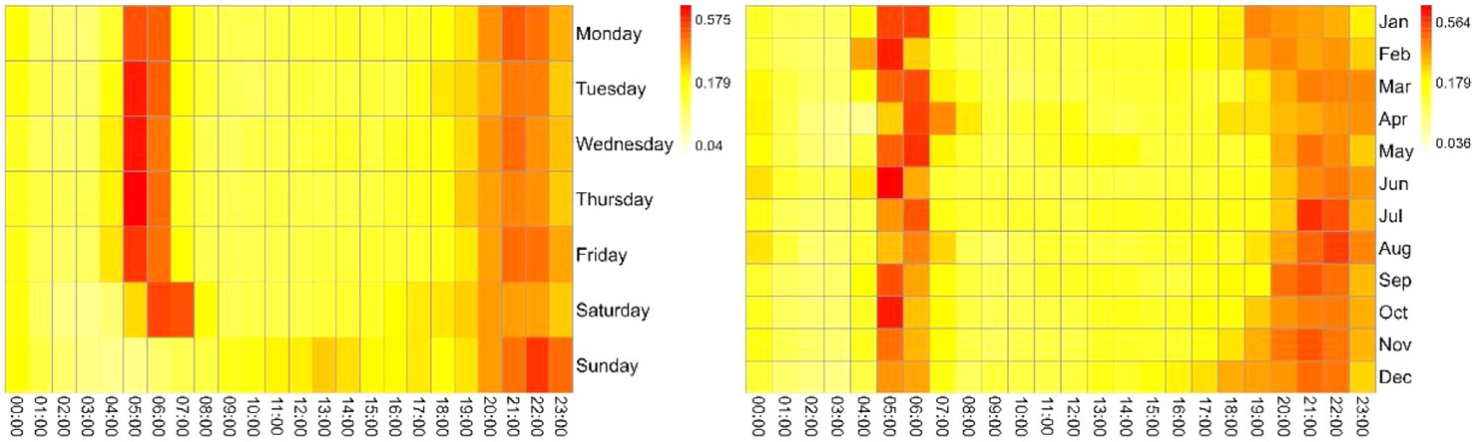
Heat Maps for electrical consumption (KWh) in household 8.

**Fig. 6:**
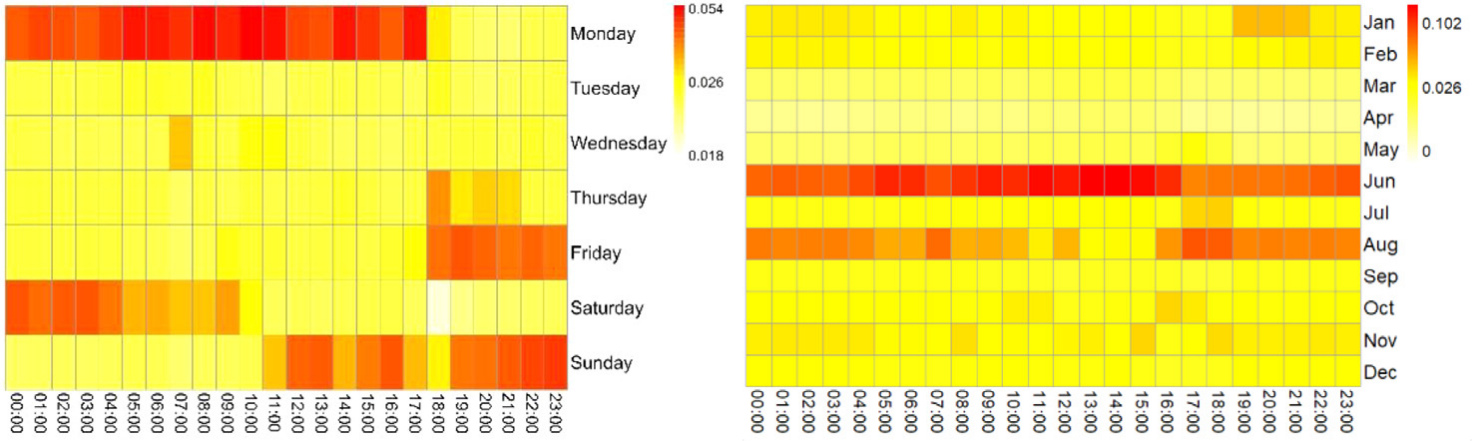
Heat Maps for electrical consumption (KWh) in household 10.

The information on electrical consumption and demand in each household is graphically represented on the heat maps of Fig. 2, Fig. 3, Fig. 4, Fig. 5, Fig. 6. Each figure contains an hourly average value of electrical consumption per weekday (left) and per month (right). This representation summarizes data that would be much more difficult to grasp if presented numerically [4], therefore it is helpful to visualize the information and to identify preliminary patterns.

### 2.4 Weather Data

The weather data presented in this article is contained inside two .csv files named *weather_outdoor variables.csv and weather_indoor variables.csv* that are found within the *weather* folder. Both files contain measurements from May 19th, 2022, to March 31st, 2023. The first file, *weather_outdoor variables.csv,* contains: Outdoor Temperature (°C), Feels Like (°C), Dew Point (°C), Wind Speed (m/s), Wind Gust (m/s), Max Daily Gust (m/s), Wind Direction (°), Hourly Rain (mm/h), Event Rain (mm), Daily Rain (mm), Weekly Rain (mm), Monthly Rain (mm), Yearly Rain (mm), Relative Pressure (hPa), Humidity (%), Ultra-Violet Radiation Index, Solar Radiation (*W/m^2^)*, Absolute Pressure (hPa), Avg Wind Direction (10 min) (°), and Avg Wind Speed (10 min) (m/s). While the second file, *weather_indoor variables.csv*, contains: Indoor Temperature (°C), Indoor Humidity (%), Indoor Feels Like (°C), and Indoor Dew Point (°C). Both files have a first column named Time, showing the date and time in which each measurement was taken using a dd/mm/yyyy HH:MM format; the names of the other columns stem from the measurements they contain.

The weather time series are given–approximately–in a 5 min resolution, yet the sampling of the measurements is not always consistent. It is important to mention that the data contains missing values, which could be more easily identified and corrected after a resampling that makes the 5 min resolution consistent. Furthermore, the dataset contains a large period of missing data starting on July 7th, 2022, at 15:35 and finishing on October 15th, 2022, at 10:41.

**Fig. 7:**
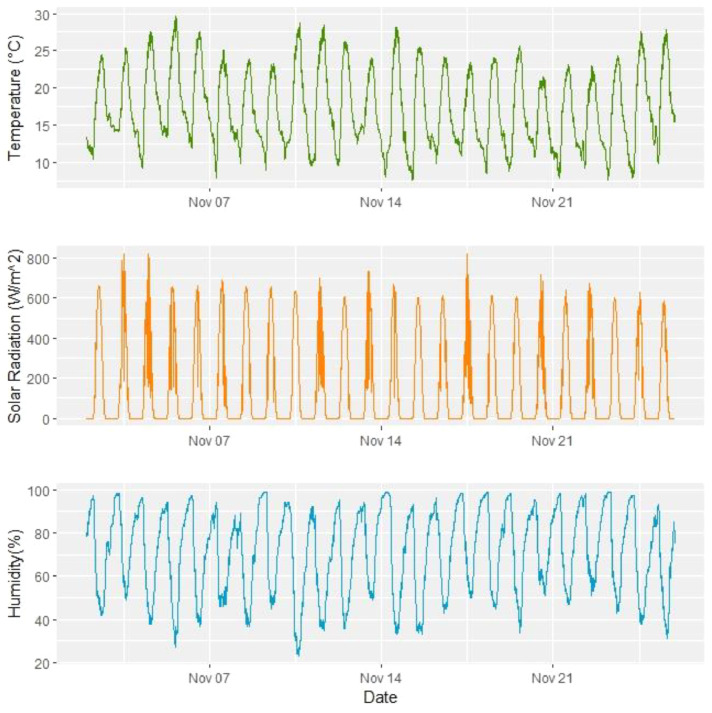
Some climatological variables for November.

To visualize part of this dataset, a sample for the Temperature, Solar radiation and Humidity are represented, in Fig. 7, for the month of November.

## 3 Experimental Design, Materials, and Methods

The *Emporia Vue Smart Energy Monitor with 50A* circuit sensors were used to measure the electrical demand and consumption of each household. These devices provide real-time measurements that can be accessed through the company's App or Website^3^. The sensor has a measurement range of 0.04 A to 75 A, with an Accuracy of±2% and a resolution of 0.04 A or approx. 5 W. The power demand and consumption in kW and kWh, respectively, is calculated from the measured values.

For the weather measurements, the *Ambient Weather WS-2000 WiFi OSPREY Solar Powered Wireless Weather Station*^4^ was used. This system consists of an outdoor measuring station and an indoor measuring unit. The station for measuring the outdoor variables was installed outside household number 7, while the sensor for measuring indoor variables was placed inside. Both were connected to that household's internet. Once connected, they uploaded the weather data to an online platform from which the data could be easily downloaded in a .csv file. Note that the outdoor measuring station was installed on top of a roof to obtain the best possible measurements without interference from surrounding buildings or objects.

Please note that the specifications for measured parameters are given in Table 3. It is important to mention that the weather values not contained in Table 3 are calculated from the ones that are measured.

**Table 3:** Weather measurement specifications taken from the products website^4^. Note that the values in the website are given in imperial units, which we converted to the SI units shown below.

Measurement	Range	Accuracy	Resolution
Indoor variables			
Temperature	-10 to 60 °C	± 1.1 °C	0.056 °C
Humidity	10 to 99%	± 5%	1%
Outdoor variables			
Temperature	-40°C to 65 °C	± 1.1 °C	0.056 °C
Humidity	10 to 99%	± 5%	1%
Pressure (barometric)	299.69 hPa to 1100.57 hPa	± 2.71 hPa	0.339 hPa
Solar Radiation	0 to 200,000 lx	± 15%	1 lx
Solar Irradiance	0 to 2367.79 W/m^2	± 15%	0.001 W/m^2
Rain	0 to 1000 mm	± 5%	0.25 mm
Wind direction	0–360°	± 10°	1°
Wind speed	0 to 44.7 m/s	± 0.983 m/s or 10% (whichever is greater)	0.626 m/s
UV radiation index	0 to 15	± 1	1

## Limitations

All the households present missing values for consumption and demand, as presented in Table 2. To make their identification easier, the missing information is labeled with a -1 in every file.

The weather time series also contains missing values for a large period starting on July 7th, 2022, at 15:35 and finishing on October 15th, 2022, at 10:41. Furthermore, the sampling of the weather data is not always consistent.

## Ethics Statement

The authors have read and follow the ethical requirements for publication in Data in Brief and confirm that the work does not involve human subjects, animal experiments or data collected from social media platforms. From the beginning, the electrical demand and consumption data was anonymized, thus the project approval committee did not consider an ethical approval necessary. Nevertheless, all participants were given an informed consent before the installation of the sensors, which they accepted and signed.

## CRediT authorship contribution statement

**Milagros Santos-Moreno:** Writing – original draft, Validation. **Jorge Ángel González-Ordiano:** Conceptualization, Writing – original draft, Supervision, Project administration, Funding acquisition. **J. Emilio Quiroz-Ibarra:** Writing – review & editing, Supervision. **D.A. Perez-DeLaMora:** Writing – review & editing, Supervision. **Jaime Mizrahi-Cojab:** Data curation. **Emilio Román-Sánchez:** Data curation. **José Pablo Montero-Cantú:** Data curation. **Lazaro Bustio-Martinez:** Writing – review & editing, Supervision.

## Data Availability

Electrical demand/consumption and climatological data of a small Mexican community (Original data) (Mendeley Data).

## Footnotes

1 https://www.mapchart.net.

## Footnotes

2 This labeling stems from the original plan of studying 10 different households; the five presented here are the ones in which the installation of the sensors was possible.

3 https://www.emporiaenergy.com/how-the-vue-energy-monitor-works.

4 https://ambientweather.com/ws-2000-smart-weather-station.
